# Activity of the novel mTOR inhibitor Torin-2 in B-precursor acute lymphoblastic leukemia and its therapeutic potential to prevent Akt reactivation

**DOI:** 10.18632/oncotarget.2490

**Published:** 2014-09-16

**Authors:** Carolina Simioni, Alice Cani, Alberto M. Martelli, Giorgio Zauli, Giovanna Tabellini, James McCubrey, Silvano Capitani, Luca M. Neri

**Affiliations:** ^1^ Department of Morphology, Surgery and Experimental Medicine, University of Ferrara, Ferrara, Italy; ^2^ Department of Biomedical and Neuromotor Sciences, University of Bologna, Bologna, Italy; ^3^ Institute for Maternal and Child Health, IRCCS “Burlo Garofolo”, Trieste, Italy; ^4^ Department of Molecular and Translational Medicine, University of Brescia, Brescia, Italy; ^5^ Department of Microbiology & Immunology, Brody School of Medicine, East Carolina University, Greenville, NC, USA; ^6^ LTTA Center, University of Ferrara, Ferrara, Italy

**Keywords:** B-pre acute lymphoblastic leukemia, Torin-2, mTOR, targeted therapy, Akt

## Abstract

The PI3K/Akt/mTOR signaling cascade is a key regulatory pathway controlling cell growth and survival, and its dysregulation is a reported feature of B-precursor acute lymphoblastic leukemia (B-pre ALL).

Torin-2 is a novel, second-generation ATP-competitive inhibitor that is potent and selective for mTOR with a superior pharmacokinetic profile to previous inhibitors. It has been shown that Torin-2 displayed dramatic antiproliferative activity across a panel of cancer cell lines.

To investigate if Torin-2 could represent a new option for the treatment of B-pre ALL, we tested its activity on a panel of B-pre ALL cell lines. In all of them Torin-2 showed a powerful cytotoxic activity, inhibiting the growth of each cell line in a dose-dependent manner, with an IC_50_ in the nanomolar range. Torin-2 caused both apoptosis and autophagy, induced cell cycle arrest in G_0_/G_1_ phase and affected both mTORC1 and mTORC2 activities as assessed by their specific substrate dephosphorylation.

Torin-2 alone suppressed feedback activation of PI3K/Akt, whereas the mTORC1 inhibitor RAD001 required the addition of the Akt inhibitor MK-2206 to achieve the same effect. These pharmacological strategies targeting PI3K/Akt/mTOR at different points of the signaling pathway cascade might represent a new promising therapeutic strategy for treatment of B-pre ALL patients.

## INTRODUCTION

mTOR is a highly conserved and widely expressed serine/threonine kinase, that is a member of the phosphatidylinositol-3 kinase–like kinase (PIKK) family, which also includes other protein kinases that regulate DNA damage responses, such as ATM (ataxia telangiectasia-mutated kinase) and ATR (ATM [ataxia telangiectasia-mutated]- and Rad3-related kinase) [1, 2].

mTOR plays a pivotal role in the PI3K/Akt/mTOR signaling pathway, which senses growth factor and serves as a central regulator of fundamental cellular processes such as cell growth/apoptosis, autophagy, translation, and metabolism [3, 4]. Activation of PI3K recruits cellular protein kinases that in turn activate downstream kinases, including the serine/threonine kinase Akt. Phosphorylation of Akt activates the mTOR complex 1 (mTORC1) and induces subsequent phosphorylation of S6K, and of the eukaryotic translation initiation factor 4E-binding protein 1 (4E-BP1). The activation of mTORC1 results in increased translation and protein synthesis [5]. A second complex of mTOR, known as mTORC2, has been more recently described and appears to act as a feedback loop via Akt [6].

Gene deletions/mutations and functional impairment of many proteins involved in this signaling pathway lead to a deregulation that results in different human cancers, including hematological malignancies. Furthermore hyperactivation of this pathway through loss of negative regulators, such as PTEN, or mutational activation of receptor tyrosine kinases upstream of phosphoinositide 3-kinase (PI3K) is a frequent occurrence in leukemia patients, where it negatively influences response to therapeutic treatments [7]. Acute lymphoblastic leukemia (ALL) is the most common pediatric malignancy and B-precursor acute lymphoblastic leukemia (B-pre ALL) is the most frequent pediatric ALL subtype, characterized by an aggressive neoplastic disorder of early lymphoid precursor cells [8, 9]. The treatment protocol for B-pre ALL includes an intense chemotherapy regimen with cure rates of 15–80% [10, 11].

In B-pre ALL many research efforts are currently devoted to the development of targeted therapies to limit side effects of chemotherapy and to increase treatment efficacy for poor prognosis patients, i.e. poor outcome following relapse [12, 13]. PI3K/Akt/mTOR pathway activation is a frequent feature in B-pre ALL [12] and therefore this pathway is an attractive target to efficiently treat this disease. A new class of ATP-competitive mTOR inhibitors, such as Torin-2, have been shown to potently target mTORC1 and mTORC2 [14]. Torin-2 is also a potent inhibitor of ATR, ATM, and DNA-PK. This compound exhibits an anti-tumour activity more broad-based and profound compared to the rapalogs that do not fully inhibit mTORC1 and are unable to inhibit mTORC2 [15].

We therefore hypothesized that dual inhibition of mTORC1 and mTORC2 by Torin-2 would provide a superior outcome in B-pre ALL as compared to inhibition of mTORC1 obtained with RAD001 [16]. We tested the cytotoxic activity of Torin-2 and its capability to prevent Akt reactivation after mTORC1 and mTORC2 inhibition. Furthermore we explored if dual targeting of mTORC1 and Akt, with RAD001 and MK-2206 respectively, might achieve results similar to those obtained with Torin-2 alone.

Torin-2 displayed a powerful cytotoxic activity with an IC_50_ in the nanomolar range, induced G_0_/G_1_ phase cell cycle arrest, modulated the PI3K/Akt/mTOR pathway and caused apoptosis and autophagy in a dose-dependent manner. Interestingly, feedback activation of PI3K/Akt was suppressed by Torin-2 alone, whereas RAD001 required the addition of MK-2206 to achieve the same efficacy. These findings indicates that mTORC1 and mTORC2 inhibition could be an attractive strategy to develop innovative therapeutic protocols for the treatment of B-pre ALL leukemia patients and to prevent Akt reactivation after mTORC1 targeting.

## RESULTS

### PI3K/Akt/mTOR pathway activation status in B-pre ALL cell lines

**Figure 1:**
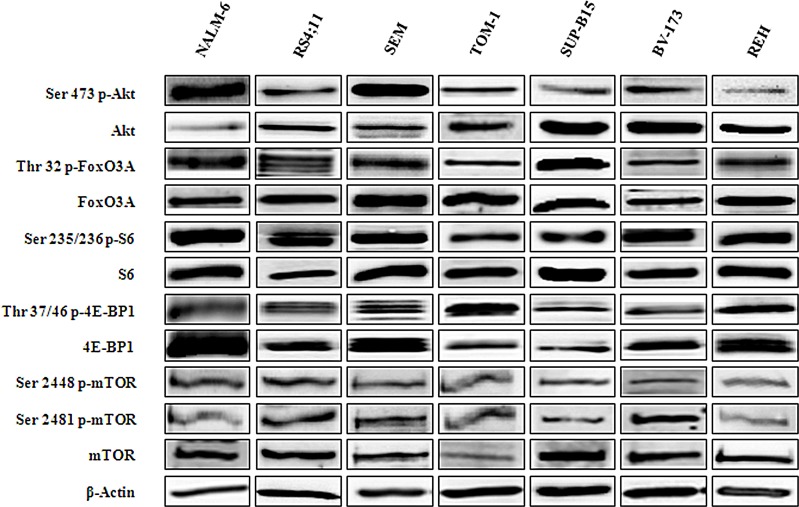
Expression and phosphorylation status of mTOR and Akt and their downstream targets in B-pre ALL cell lines Western blot analysis of B-pre ALL cell lines to detect the expression and phosphorylation levels of Akt, mTOR and its downstream substrates. Twenty-five μg of protein were blotted to each lane. Antibody to β-actin served as a loading control.

We first analyzed by Western blot the baseline expression of key components of the PI3K/Akt/mTOR pathway and their phosphorylation status in a panel of human B-pre ALL cell lines (NALM-6, SEM, REH, RS4;11, BV-173, SUP-B15, TOM-1). Three of these cell lines, BV-173, SUP-B15 and TOM-1 are Ph^+^, since they harbour the Bcr-Abl fusion protein. Despite some heterogeneity, all the B-pre ALL cell lines displayed phosphorylation at the Ser 2448 and Ser 2481 (readout for mTORC1 and mTORC2, respectively) residues of mTOR and at the Ser 473 residue of Akt, which are indicative of the constitutive activation of this signaling pathway (Fig. 1).

We further explored the basal condition of downstream targets of both kinases. In agreement with the hyperactivated status of mTORC1, the ribosomal protein S6 and the eukaryotic translation initiation factor 4E-binding protein 1 (4E-BP1) were phosphorylated at Ser 235/236 and Thr 37/46 respectively (Fig. 1). A readout for mTORC2 activity is represented by the phosphorylation at the Ser 473 site of Akt. Hyperactivation of Akt resulted in the phosphorylation at the Thr 32 site of Forkhead box O3A (FoxO3A) (Fig. 1).

### Torin-2 induces cytotoxicity, blocks cell cycle progression at the G_0_/G_1_ phase and induces autophagy

**Figure 2:**
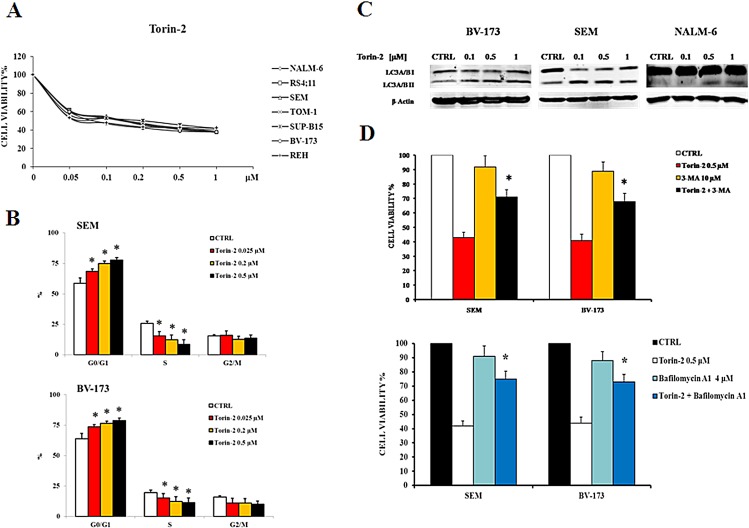
Torin-2 is cytotoxic, cytostatic and induces autophagy in B-pre ALL cell lines A. MTT assay of B-pre ALL cell lines treated with increasing concentrations of Torin-2 for 48h. One representative experiment is shown. B. Flow cytometric analysis for SEM and BV-173 cells treated with increasing concentrations of Torin-2 for 24h. Asterisks indicate statistically significant differences with respect to untreated cells (*p<0.05). Cell lines displayed are representative of the cell panel used in this study. C. Effect of Torin-2 on autophagy in BV-173, SEM and NALM-6 cells, documented by the lipidation of the autophagy marker LC3A/B. Antibody to β-actin served as a loading control. D. The activity of 3-MA in combination with Torin-2 is reported in the upper histogram, after SEM and BV-173 treatment for 24h. Below, the effect on cell viability of SEM and BV-173 cells after treatment with Torin-2 and the autophagy inhibitor Bafilomycin A1 is reported. Results are the mean of three different experiments ± SD. Asterisks indicate statistically significant differences with respect to untreated cells (*p<0.05).

To determine whether Torin-2 could affect viability of B-pre ALL cell lines, cells were incubated in the presence of increasing concentrations of Torin-2 for 48h and then analyzed by MTT assays. All cell lines resulted sensitive to the drug, in a concentration-dependent fashion, with the IC_50_ value that ranged between 0.07 μM and 0.19 μM (Fig. 2A). Given the importance of the PI3K/Akt/mTOR signaling pathway in the regulation of cell proliferation, the effects of Torin-2 on cell cycle progression were also investigated. SEM and BV-173 cell lines were treated with increasing concentrations of Torin-2 for 24h, then cells were harvested, fixed and stained with Propidium Iodide (PI) and analyzed with the Muse^TM^ Cell Analyzer. The assay showed in both cell lines a concentration-dependent increase of G_0_/G_1_ phase of cell cycle and a simultaneous decrease in the S phase (Fig. 2B).

Autophagy can be a form of programmed cell death, but is also involved in protective mechanisms against apoptosis [17, 18]. To evaluate whether the treatment with Torin-2 could lead to autophagy, we detected the expression of LC3A/B I (non lipidated) and LC3A/B II (lipidated) by Western blot in BV-173, SEM and NALM-6 cells treated with increasing concentrations of Torin-2. The expression levels of LC3A/B II gradually increased in the three cell lines in a dose-dependent manner (Fig. 2C).

To verify whether autophagy was either a cell survival or a cell death mechanism, we used the autophagy inhibitor 3-MA (3-Methyladenine), which blocks an early stage of autophagy by inhibiting the class III phosphoinositide 3-kinase (PI3K) [19]. 3-MA alone did not affect cell growth, even at the concentration of 10 μM (cell viability was comparable to untreated cells), but cells treated with 3-MA become significantly more resistant to Torin-2 cytotoxic effect (Fig. 2D, upper panel). We also employed Bafilomycin A1, another well established autophagy inhibitor that inhibits vacuolar ATPase (V-ATPase) and promotes the accumulation of autophagic vacuoles [20]. As shown in the lower panel of Fig. 2D, the treatment with 4 μM Bafilomycin A1 confirmed that inhibition of autophagy significantly reduced the cytotoxic effect of Torin-2. These results indicated that autophagy is a critical determinant of the cytotoxic effects induced in B-pre ALL cells by Torin-2.

### Torin-2 causes pro-apoptotic effects on B-pre ALL cell lines

**Figure 3:**
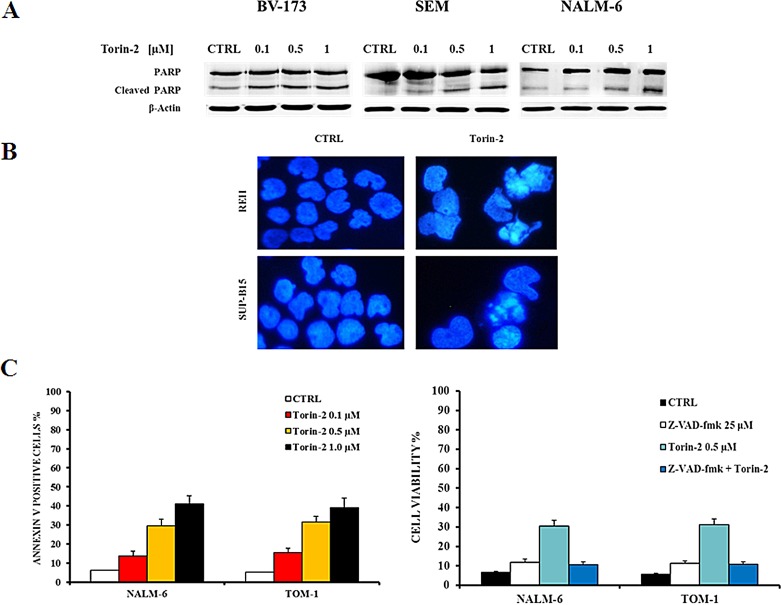
Torin-2 induces apoptosis A. Western blot analysis documenting a Torin-2 concentration-dependent PARP cleavage in BV-173, SEM and NALM-6 cells. Antibody to β-actin served as a loading control. B. DNA staining of REH and SUP-B15 with the fluorescent dye DAPI is reported. In REH and SUP-B15 treated with 0.5 μM Torin-2 various aspects of nuclear shrinkage, fragmentation and chromatin margination, that are associated with the apoptotic mode of cell death, are observable. C. On the left, flow cytometric analysis of NALM-6 and TOM-1 cell lines treated with increasing concentrations of Torin-2. Samples were incubated with Annexin V-fluorescein isothiocyanate. On the right, MTT assay of NALM-6 and TOM-1 cells treated with Torin-2 and Z-VAD-fmk, a pan caspase inhibitor, is showed. Results are the mean of three different experiments ± SD.

To investigate whether the decreased viability was related to apoptosis, cells were treated for 24h with increasing concentrations of Torin-2 and analyzed by both Western blot and DNA staining. Cleavage of poly(ADP-ribose) polymerase (PARP) in BV-173, SEM and NALM-6 revealed the pro-apoptotic effect of Torin-2. REH and SUP-B15 cells were treated with Torin-2 at 0.5 μM for 24h (Fig. 3A). DAPI staining revealed the morphological changes associated with apoptosis, such as chromatin condensation and nuclear fragmentation (Fig.3B). Apoptosis was further investigated by flow cytometric analysis of Annexin V-stained samples, that showed in NALM-6 and TOM-1 a significant and concentration-dependent increase of apoptotic cells (Fig. 3C, left panel). To determine whether activated caspases are involved in the apoptotic action of Torin-2, we examined the effects of the broad spectrum caspase inhibitor Z-VAD-fmk on cell apoptosis as determined by annexin-V FITC binding. Torin-2-mediated apoptosis was blocked markedly by 25 μM Z-VAD-fmk (Figure 3C, right panel). Therefore these results showed that Torin-2 induced caspase-dependent cell death.

### Torin-2 affects the PI3K/Akt/mTOR pathway in B-pre ALL cells

**Figure 4:**
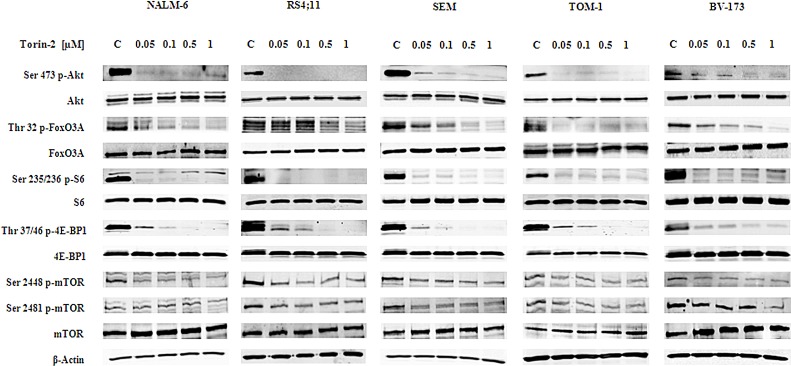
Torin-2 cytotoxicity is related to PI3K/Akt/mTOR signaling pathway inhibition Western blot analysis for mTOR, Akt and their downstream targets S6, 4E-BP1 and FoxO3A in B-pre ALL cell lines. Twenty-five μg of protein were blotted to each lane. In all samples 2h of Torin-2 treatment with increasing concentrations was performed. β-actin served as a loading control.

To assess the effects of Torin-2 on the PI3K/Akt/mTOR signaling pathway, we studied the expression and activation status of critical components of the PI3K/Akt/mTOR cascade. NALM-6, RS4;11, SEM, TOM-1 and BV-173 cells were treated with increasing concentrations of Torin-2 for 2h and Western blot was then performed (Fig. 4).

Torin-2 decreased the phosphorylation levels of mTOR on both the Ser 2448 and Ser 2481 residues. It should be remembered that the phosphorylation of mTOR on Ser 2481 is a mTORC2-selective autophosphorylation site [21]. mTORC1 inhibition had functional effects on two well known mTORC1 substrates, S6 and 4E-BP1. S6 was completely dephosphorylated on the Ser 235/236 residue already at 50 nM concentration of Torin-2 in all cell lines, whereas 4E-BP1 was fully dephosphorylated on the Thr 37/46 site starting from the 100 nM concentration. Total levels of all these proteins were instead unaffected by Torin-2.

mTORC2 inhibition had a readout in Ser 473 Akt dephosphorylation and it was observable in all the cell lines starting from Torin-2 concentration of 50 nM. Despite some differences, also the Akt downstream substrate FoxO3A was dephosphorylated in a dose dependent fashion on its Thr 32 residue in all cell lines (Fig. 4).

### Torin-2 prevents the reactivation of Akt upon mTOR inhibition in B-pre ALL cells

Since it has been previously described that in hematological malignancies [22, 23] and solid tumors [24, 25] with constitutive PI3K/Akt activation, the rapamycin derivative inhibitor everolimus (RAD001) increased Akt phosphorylation, we sought to explore if Torin-2 might prevent Akt re-activation after mTORC1 inhibition.

**Figure 5:**
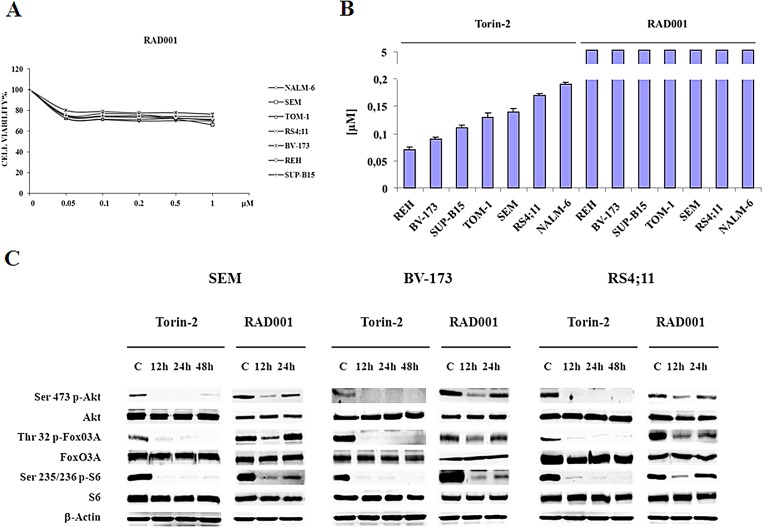
Torin-2 prevents Akt reactivation in B-pre ALL A. MTT assay of B-pre ALL cell lines treated with increasing concentrations of RAD001 for 48h. One representative experiment of three is shown. B. IC_50_ values for Torin-2 and RAD001 on the viability of B-pre ALL cell lines after 48h. Results are the mean of three different experiments ± SD. C. Western blot analysis for PI3K/Akt/mTOR signaling pathway in SEM, BV-173 and RS4;11 cells. Cells were treated with 0.15 μM Torin-2 and 0.6 μM RAD001 for different times of incubation. In RAD001 treated samples after 24h is evident the re-phosphorylation of Akt, FoxO3A and S6. β-actin served as a loading control.

For this set of experiments we decided to use the concentration of 0.15 μM, that represents nearly the average IC_50_ of Torin-2 in the panel of cell lines employed. We prolonged the treatment with Torin-2 up to 48h and we compared it with the mTORC1 inhibitor, RAD001, employed at the concentration of 0.6 μM. This value has been chosen to mirror a concentration of a mTORC1 inhibitor (Temsirolimus) correspondent to the plasma concentration achievable in clinical trials [26]. As shown in Fig. 5A and B, in a range from 0.05 to 5 μM, RAD001 alone could not achieve the IC_50_. Thus, RAD001 had a putative IC_50_ higher than 5 μM, whereas Torin-2 displayed an IC_50_ value < 0.2 μM in all cell lines. Interestingly, we found that either mTORC1 and mTORC2 substrates, including Akt, after 48h of Torin-2 treatment remained dephosphorylated (Fig. 5C). On the contrary, samples treated with RAD001 already after 24h showed a re-phosphorylation of Akt. Direct (FoxO3A) or indirect (S6) downstream targets of Akt displayed the same behaviour (Fig. 5C).

### The allosteric Akt inhibitor MK-2206 synergizes with RAD001 but not with Torin-2

For therapeutic targeting of the PI3K/Akt/mTOR pathway, the combined inhibition at different points of the cascade often leads to more effective results than the use of a drug that acts on a single or dual targets [27]. To better assess the potential therapeutic value of Torin-2 in B-pre ALL, we analyzed its synergistic potential with MK-2206, an orally active, allosteric Akt inhibitor, which is currently tested in phase II clinical trials. We also compared this drug combination with a second one consisting of the association of MK-2206 and RAD001. The drugs were used at a fixed ratio (1:1 both for Torin-2/MK-2206 and RAD001/MK-2206).

**Figure 6:**
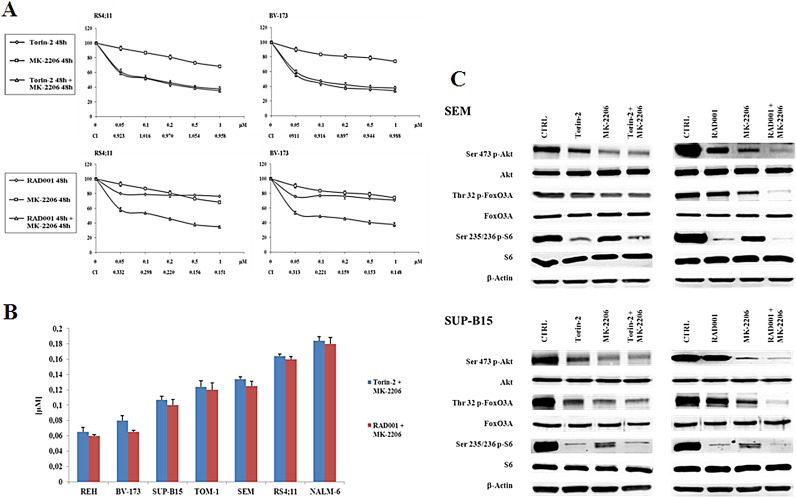
Dual administration of Torin-2 or RAD001 with MK-2206 in B-pre ALL cell lines A: B-pre ALL cell lines were treated for 48h with Torin-2 or RAD001, either alone or in combination with MK-2206. Results are the mean of three different experiments ± SD. Combination index (CI) value for each data point was calculated with the appropriate software for dose effect analysis (Calcusyn). B: IC_50_ values for Torin-2 or RAD001 in combination with MK-2206 on the viability of B-pre ALL cell lines at 48h. Results are the mean of three different experiments ± SD. C: Western blot analysis for PI3K/Akt/mTOR in SEM and SUP-B15 cells. Cells were treated for 30 minutes with 0.15 μM Torin-2 or 0.6 μM RAD001 in combination with 0.5 μM MK-2206. β-actin served as a loading control.

After 48h of treatment, MTT assays were performed. The dual targeting of mTORC1/mTORC2 and Akt with Torin-2 and MK-2206 did not show a synergistic effect at any concentration, whereas the administration of RAD001 and MK-2206 together resulted in a relevant synergistic cytotoxic effect in RS4;11 and BV-173 cell lines (Fig. 6A). This phenomenon was more relevant in the range between 0.5 and 1 μM, as confirmed by the combination index (CI) values. Similar results were obtained with other B-pre ALL cell lines (supplementary Fig. 1). It should be noted that a comparison between the IC_50_ values obtained from the two different treatment at 48h (Torin-2/MK-2206 and RAD001/MK-2206), showed that the IC_50_ values were very similar and comparable in each cell line (Fig. 6B).

We next studied the effects of the two drug combinations on the phosphorylation levels of Akt, FoxO3A and S6 protein. Torin-2 was used at 0.15 μM, RAD001 at 0.6 μM and MK-2206 at 0.5 μM for 30 minutes and then Western blot analysis was performed.

**Figure 7:**
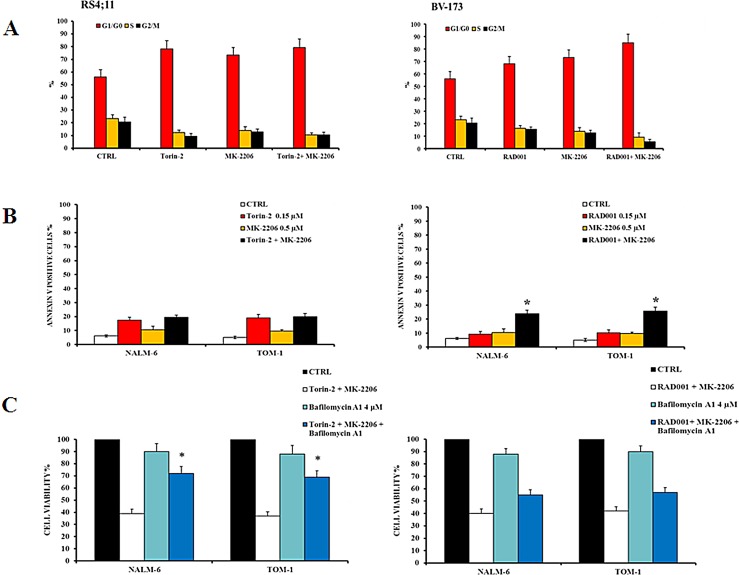
Effects of dual administration of Torin-2 or RAD001 with MK-2206 in B-pre ALL cell lines A: Flow cytometric representation of the effects of Torin-2/MK-2206 and RAD001/MK-2206 combinations on the cell cycle of RS4;11 and BV-173 cell lines after 24h of treatment. Results are the mean of three different experiments ± SD. B: Flow cytometric analysis of NALM-6 and TOM-1 cell lines treated with increasing concentrations of Torin-2 or RAD001, alone and in combination with MK-2206, for 24h. Samples were incubated with Annexin V-fluorescein isothiocyanate and then analyzed for apoptosis. Results are the mean of three different experiments ± SD. Asterisks indicate statistically significant differences with respect to untreated cells (*p<0.05). C. MTT assay representation of the autophagy effects on NALM-6 and TOM-1 cells treated with Torin-2/MK-2206 or RAD001/MK-2206 combinations or plus the administration of the autophagy inhibitor Bafilomycin A1. Results are the mean of three different experiments ± SD. Asterisks indicate statistically significant differences with respect to untreated cells (*p<0.05).

The concentration of MK-2206 was selected for either being in the range of maximal synergy and to be comparable to the plasma concentration that has been obtained in clinical trials in acute myelogenous leukemia [28]. Both SEM and SUP-B15 cell lines did not show a synergistic effect of Torin-2 and MK-2206 on the phosphorylation levels of Akt, FoxO3A and S6. Interestingly, there was an additional downregulation of protein phosphorylation only when the dual treatment RAD001/MK-2206 was administered, thus confirming the synergistic effect in modulating the PI3K/Akt/mTOR pathway (Fig. 6C). To further assess these findings, we explored the effects of dual treatments on cell cycle, by flow cytometric analysis of PI-stained samples in RS4;11 and BV-173 cells cultured for 24h. Both drug associations yielded comparable results. The treatment with Torin-2 and MK-2206 was similar to the single administration of Torin-2 alone. At variance, the dual administration of RAD001 and MK-2206 increased the percentage of cells in the G_0_/G_1_ phase of the cell cycle in comparison with single drug administration (Fig. 7A).

We also examined the pro-apoptotic effect of the two drug association on NALM-6 and TOM-1 cells. Cells were treated with 0.15 μM Torin-2 or 0.6 μM RAD001 and 0.5 μM MK-2206 for 24h and then analyzed by MTT assays. Using these drug concentrations, nearly 20% of apoptotic cells were observed in either cell line with both drug combinations. However, apoptosis induced by the treatment with Torin-2 and MK-2206 was comparable with that observable with the administration of Torin-2 alone, whereas the apoptosis obtained with the dual administration of RAD001 and MK-2206 was the consequence of a synergistic effect (Fig. 7B).

Finally, the effect of Bafilomycin A1 on dual targeting the PI3K/Akt/mTOR pathway with a combination of Torin-2/MK-2206 or RAD001/MK-2206 was analyzed. A consistent inhibition of autophagy increased the viability of B-pre ALL cells treated with Torin-2/MK-2206 (Fig. 7C, left panel) and to a lower extent with RAD001/MK-2206 (Fig. 7C, right panel). This difference may rely on the different mechanism through which autophagy is recruited by the drugs: those who act on mTOR, activated autophagy as a cell death mechanism [16],whereas the drug that inhibit Akt, induced autophagy as a cell protection mechanism [29].

### Torin-2 suppresses doxorubicin-induced cell cycle checkpoint activation

When DNA is damaged by DNA intercalating agents, such as Doxorubicin, double stranded breaks (DSBs) trigger recruitment of ATM and ATR to the damage site which in turn phosphorylates histone H2AX leading to foci formation [30].

Torin-2 also exhibited potent biochemical and cellular activity against PIKK family kinases including ATM, ATR, and DNA-PK, whose inhibition sensitized cells to irradiation [15]. The ATR pathway is known to transmit DNA damage signals through the ATR-CHK1 kinase cascade and activation of cell cycle checkpoint regulators such as CHK1 and CHK2 has a critical role in promoting cell cycle arrest in response to cytotoxic agents, including doxorubicin [31].

**Figure 8:**
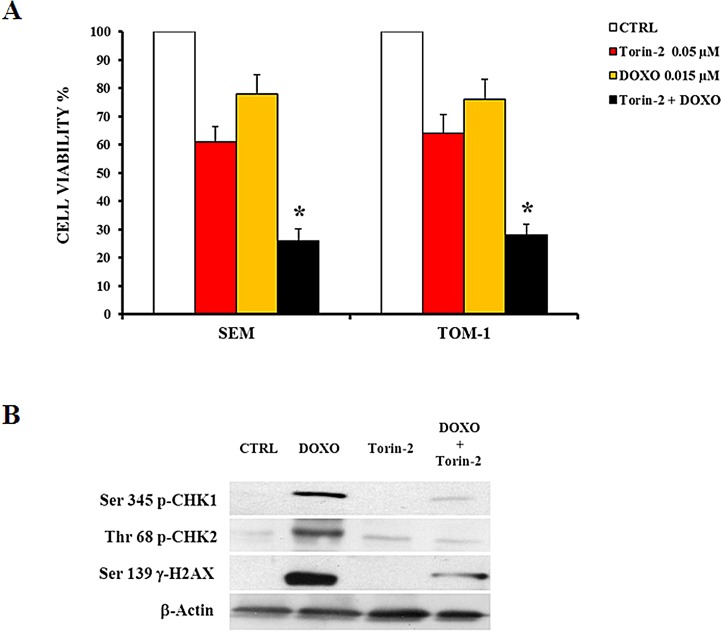
Torin2 suppresses Doxorubicin-induced cell cycle checkpoint activation A: MTT assay showing the cytotoxic effect of Torin-2 and Doxorubicin (0.015 μM) alone or in combination in SEM and TOM-1 cells. DOXO, Doxorubicin-treated cells. Results are the mean of three different experiments ± SD. Asterisks indicate statistically significant differences with respect to untreated cells (*p<0.05). B: Western blot analysis of SEM cells showing the activation by Doxorubicin treatment of cell cycle checkpoint regulators Chk1 and Chk2, markers for ATR or ATM activity, respectively. Torin-2 alone did not modify CHK1 and CHK2 phosphorylation, whereas it dramatically decreased after Doxorubicin administration. Torin-2 also influenced the levels of phosphorylated γ-H2AX, inhibiting its phosphorylation after Doxorubicin treatment. β-actin served as a loading control.

We tested if the combination of Doxorubicin and Torin-2 may exert additional cytotoxic activity than the two drugs administered alone. In Figure 8A are shown the results of MTT assays of two representative cell lines (SEM and TOM-1) analyzed for cell viability after treatment with the drugs used either as single agents or combined together. In both cell lines, the drug combination induced a stronger decrease in cell viability (Fig. 8A). Doxorubicin alone induced phosphorylation of CHK1 (Ser 345, a marker for ATR activity) and CHK2 (Thr 68, a readout for ATM activity). In contrast, Torin-2 (0.25 μM) alone did not increase CHK1 and CHK2 phosphorylation, whereas it dramatically decreased the phosphorylation induced by Doxorubicin (Fig. 8B). One prominent chromatin modification in response to DNA damage is phosphorylation of histone H2AX on Ser 139, which is referred to as γ-H2AX [32].

Torin-2 inhibited the DNA damage response induced by Doxorubicin, as documented by the effects on the levels of phosphorylated γ-H2AX on the Ser 139 residue (Fig. 8B).

## DISCUSSION

Owing to the fundamental role of PI3K/Akt/mTOR pathway in tumor development and progression, there has been a significant interest in developing inhibitors against components of this pathway, up to having now many compounds currently under evaluation in clinical trials. ATP-competitive mTOR kinase inhibitors represent a promising new approach to target the PI3K/Akt/mTOR pathway with potentially greater tolerability than dual PI3K/mTOR inhibitors [5, 33, 34]. It has been previously reported that first generation mTOR kinase inhibitors had improved efficacy compared to rapamycin in models of Ph+ B-ALL [35].

We have recently explored the therapeutic potential of RAD001, an allosteric mTORC1 inhibitor in pre-clinical models of B-pre ALL [16]. We documented that RAD001 decreased cell viability, induced cell cycle arrest in G_0_/G_1_ phase and caused apoptosis in B-pre ALL cell lines. Autophagy was also induced, which was important for the RAD001 cytotoxic effect, as downregulation of Beclin-1 reduced drug cytotoxicity. RAD001, used in the micromolar range and administered 24h before MK-2206 showed the capacity to synergize with MK-2206 in both cell lines and patient samples [16]. In this study, we evaluated for the first time the efficacy of the novel mTORC1/mTORC2 second generation, ATP-competitive inhibitor, Torin-2 [14] in pre-clinical settings of Ph^+^ and Ph^−^ B-pre ALL.

Torin-2 was both cytotoxic and cytostatic in a nanomolar range to B-pre ALL cell lines in a concentration dependent mechanism, as demonstrated by MTT assays, flow cytometric analysis of Annexin V-stained samples and of PI-stained samples. Apoptosis resulted to play a determinat role in the killing mechanism, since the treatment with a pan caspase inhibitor protected the cells from Torin-2 cytotoxic effect. These results are in agreement with those observed with other drugs in acute myeloid leukemia [36, 37].

Torin-2 also induced autophagy, as documented by increased expression of the lipidated form of LC3. In order to demonstrate whether autophagy was either a survival or a death mechanism, B-pre ALL were co-treated with the autophagic sequestration inhibitors, 3-MA and Bafilomycin A1. We found that either treatment with 3-MA and Bafilomycin A1, resulted in a lower sensitivity of both SEM and BV-173 cells to the cytotoxic effects of RAD001 and indicated that Torin-2-induced autophagy was very important for the cytotoxic effects of the drug. The phosphorylation status of the key elements of the PI3K/Akt/mTOR pathway, assessed by Western blot, was equally sensitive to Torin-2 inhibition in either cells harboring or not the Bcr-Abl fusion protein.

RAD001, when used in the nanomolar range against this panel of cell lines, was much less cytotoxic than Torin-2, as it displayed IC_50_s > 5 μM. This superior efficacy of the second generation inhibitors, has been also reported for MLN0128, another mTORC1/mTORC2 inhibitor, which displayed an improved anti-leukemic activity when compared to first generation inhibitors, in Ph^−^ B-ALL derived from both adult and pediatric subjects [21]. It has been reported that in solid tumor models, activation of mTORC1 drives p70S6K-mediated degradation of the IR/IGF-1R adaptor protein IRS-1, and is therefore a negative regulator of PI3K [38]. Accordingly, drugs targeting mTORC1 block this feedback and trigger reactivation of the pathway and re-phosphorylation of Akt on Ser 473 residue in acute myelogenous leukemia cells [22, 39]. The issue of Akt reactivation in response to mTORC1 inhibition, has not been throughly investigated in B-pre ALL. It is worth highlighting that Torin-2 maintained a prolonged suppression of mTORC1/mTORC2 with a sustained anti-proliferative effect, overcoming the limitations of rapalogs such as RAD001, as demonstrated by our findings, which resulted in re/hyper-phosphorylation of Akt and could hamper their anti-tumor action and enhance resistance to antineoplastic therapy, thus resulting in a poor outcome [40].

Akt activity is directly downregulated on Thr 308 by the protein phosphatase PP2A [41], which is also critically involved in regulation of cell cycle progression [42] and DNA damage response [43]. Accumulating evidence indicates that PP2A acts as a tumor suppressor and impairment of PP2A activity may result in loss of this function [7]. The capacity of Torin-2, but not of RAD001, to inhibit both mTORC1 and 2 may represent a possible mechanism for why Akt is not re-phosphorylated with this drug on the Ser 473 residue and this phosphorylation may be independent form PP2A activity.

The Torin-2 prolonged suppression of Akt phosphorylation may overcome the occurrence of PP2A oncosuppressor altered function and may render Akt inhibition independent from the activity of PP2A, thus proposing this therapeutic options as a potential tool that could act functionally on either impaired Akt and PP2A functions.

Overall, Torin-2 alone was as potent as a combination consisting of RAD001 and MK-2206, in terms of reduction of cell viability, apoptosis induction, and cell cycle block. In contrast, when MK-2206 was combined with Torin-2, it did not display any synergistic effect. Interestingly, the presence of synergism or its lack was evident also when the phosphorylation status of key elements of the pathway was analyzed by Western blot.

Both ATM and ATR play a central role in coordinating the DNA damage response, including cell cycle checkpoint control and apoptosis [44]. Since the results by Liu et al. [15], using purified enzymes, suggested that Torin-2 was also an inhibitor of several PIKK family members including ATR, ATM, and DNA-PK, we investigated if this was also true in B-pre ALL cell lines. Western blot analysis documented that indeed Torin-2 inhibited ATM and ATR in intact cells and, by inhibiting DNA repair, potentiated the cytotoxic effect of Doxorubicin. Torin-2 displayed an obvious advantage over RAD001, which also induced a similar phenomenon, as Torin-2 could be used in the nanomolar range, whereas RAD001 required a concentration as high as 16 μM [45], which could not be attained *in vivo*. In addition, very recently it has been reported that Akt inhibition with CCT12893 increased the phosphorylation of CHK1/CHK2 and γ-H2AX [46].

These findings open a new very interesting field to be further explored in the future regarding the therapeutic effect of PI3K/Akt/mTOR inhibitors involving DNA damage sensors and cell cycle checkpoints such as CHK1 and CHK2.

In conclusion, our data indicate that the novel mTORC1/mTORC2 kinase inhibitor Torin-2 can suppress the growth of both Ph^+^ and Ph^−^ B-pre ALL cells and extend the finding that the antiproliferative and proapoptotic effects of PI3K/Akt/mTOR pathway inhibitors are independent from ABL-translocation, as reported in long-term cultures of Ph^+^ and Ph^−^ B-precursor ALL cells from patients [40]. Remarkably, Torin-2, even after a 48h incubation, blocked the reactivation of Akt, thus confirming the new therapeutic hopes that this second generation of inhibitors is developing. In addition, Torin-2 could be also effective in combination with chemotherapeutic DNA-damaging agents, in light of its capacity of blocking DNA repair.

Interestingly, with the aim of improving B-pre ALL treatment, we also came to the conclusion that low concentrations of RAD001 and MK-2206 (which can be attained *in vivo*) may achieve a therapeutic efficacy comparable to an mTORC1/mTORC2 inhibitor. These therapeutic strategies could be particularly effective when combined with targeted next generation sequencing of tumor samples, since genomic alterations can be detected and help to identify refractory patients with aberrations putatively activating the PI3K/Akt/mTOR pathway [47, 48]. These pharmacological options targeting PI3K/Akt/mTOR at different points of the signaling pathway cascade or in combination with conventional chemotherapy might represent a new therapeutic potential for treatment of B-pre ALL patients.

## MATERIALS AND METHODS

### Materials

Alpha-MEM, McCoy’s 5A and RPMI-1640 mediums, fetal bovin serum (FBS), penicillin and streptomycin were from Lonza (Lonza Milano SRL, Milan, Italy). Torin-2, RAD001, and MK-2206 were from Selleck Chemicals (Houston, TX, USA). For cell viability determination, Cell Proliferation Kit I (MTT) was purchased from Roche Applied Science (Basel, Switzerland). Annexin V/7-AAD detection kit was from Merck-Millipore (Darmstadt, Germany). Akt-1, Ser 473 p-Akt-1, and FoxO3A primary antibodies were from Santa Cruz Biotechnology (Santa Cruz, CA, USA) while all the other antibodies were from Cell Signaling Technology (Danvers, MA, USA), including the rabbit secondary antibody. The mouse secondary antibody, Bafilomycin A1, Z-VAD-fmk, 3-Methyladenine (3-MA), 1,4-Diazabicyclo[2.2.2]octane (DABCO) and 4′, 6 diamidino-2-pheny-lindole (DAPI) were from Sigma Aldrich (Milan, Italy). Signals were detected with the ECL Plus reagent purchased from Perkin Elmer (Boston, MA, USA).

### Cell culture and Western blot analysis

All the B-pre acute lymphoblastic leukemia cell lines were obtained from Deutsche Sammlung von Mikroorganismen und Zellkulturen GmbH (Braunschweig, Germany). SEM, REH, BV-173 and NALM6 were grown in RPMI 1640 medium supplemented with 10% heat-inactivated fetal bovine serum (FBS); RS4;11 cells were grown in Alpha-MEM medium with 10% FBS; TOM-1 cells were grown in RPMI 1640 medium with 20% FBS and SUP-B15 cells were grown in McCoy’s 5A medium with 20% FBS. All the media were supplemented with 100 units/ml penicillin and 100 mg/ml streptomycin. The cells were grown at a density of 0.5 to 2 × 10^6^ cells/ml and were incubated at 37°C with 5% CO_2_. Western blot analysis was performed by standard methods as described elsewhere [49].

### Cell viability analysis

MTT (3-(4,5-dimethylthythiazol-2-yl)-2,5-diphenyltetrazolium bromide) assays were performed as previously described [50].

### Cell cycle analysis

Cell cycle analysis was performed using the Muse^TM^ Cell Analyzer (Merck Millipore, Milan, Italy) and/or propidium iodide (PI)/RNase A staining by flow cytometry according to standard techniques, as described elsewhere [51]. In brief, after 24h of drug treatment, cells were harvested, centrifuged at 300 x g for 5 min and washed once with 1X PBS. After fixing them with 70% ethanol at 20°C, cells were centrifuged at 300 x g for 5 min and washed once with 1X PBS. Then 200 μl of Muse^TM^ Cell Cycle reagent or 100 μl of propidium iodide (PI)/RNase A staining was added to each tube with an incubation of 30 min at room temperature in the dark. Samples were then analyzed according to the manufacturer’s instructions.

### PI/Annexin V assay

Apoptosis analysis was performed by staining with Annexin V/7-AAD, using the Muse^TM^ Cell Analyzer in according to the manufacturer’s instructions. In brief, a 100 μl treated cell suspension was labeled for 20 min in the dark with the same volume of the Muse^TM^ Annexin-V & Dead Cell reagent (Merck Millipore). Subsequently, quantitative detection of Annexin-V/7-AAD positive cells was performed with the Muse^TM^ Cell Analyzer [51].

### DAPI staining

Cell nuclear morphology was evaluated by fluorescence microscopy following DAPI staining. Cells were treated with Torin-2 for 24 h. The cells were washed with PBS (pH 7.4), cytocentrifuged, fixed with 4% paraformaldehyde/PBS and stained for 3 min with 1 μg/mL DAPI. The cells were then washed with PBS, specimens were embedded in glycerol with antifading agent (DABCO) and examined under Zeiss Axiophot fluorescence microscope (Zeiss, Germany).

### Combined drug effect analysis

The combination effect and the potential synergy of Torin-2 and RAD001 with MK-2206 were evaluated from quantitative analysis of dose–effect relationship, as described previously [16]. For each Torin-2/MK-2206 or RAD001/MK-2206 combination experiment, a combination index (CI) number was calculated using the Biosoft CalcuSyn software (Biosoft, Cambridge, UK). This method of analysis generally defines CI values from 0.9 to 1.1 as additive, from 0.3 to 0.9 as synergistic and 0.3 as strongly synergistic, whereas values over 1.1 are considered as antagonistic.

### Statistical evaluation

The data are presented as mean values from three separate experiments ± s.d. Data were statistically analyzed by a Dunnet test after one-way analysis of variance (ANOVA) at a level of significance of P<0.05 vs control samples.

## SUPPLEMENTARY FIGURE


