# Supplementary material for: Activity of the novel mTOR inhibitor Torin-2 in B-precursor acute lymphoblastic leukemia and its therapeutic potential to prevent Akt reactivation
Source: Oncotarget. 2014 Sep 16;5(20):10034–47. doi: 10.18632/oncotarget.2490 (PMC4259403; doi:10.18632/oncotarget.2490)
Supplement: Supplementary file 1 [file oncotarget-05-10034-s001.pdf]

Activity of the novel mTOR inhibitor Torin-2 in B-precursor acute lymphoblastic leukemia and its therapeutic potential to prevent Akt reactivation

Supplementary Material

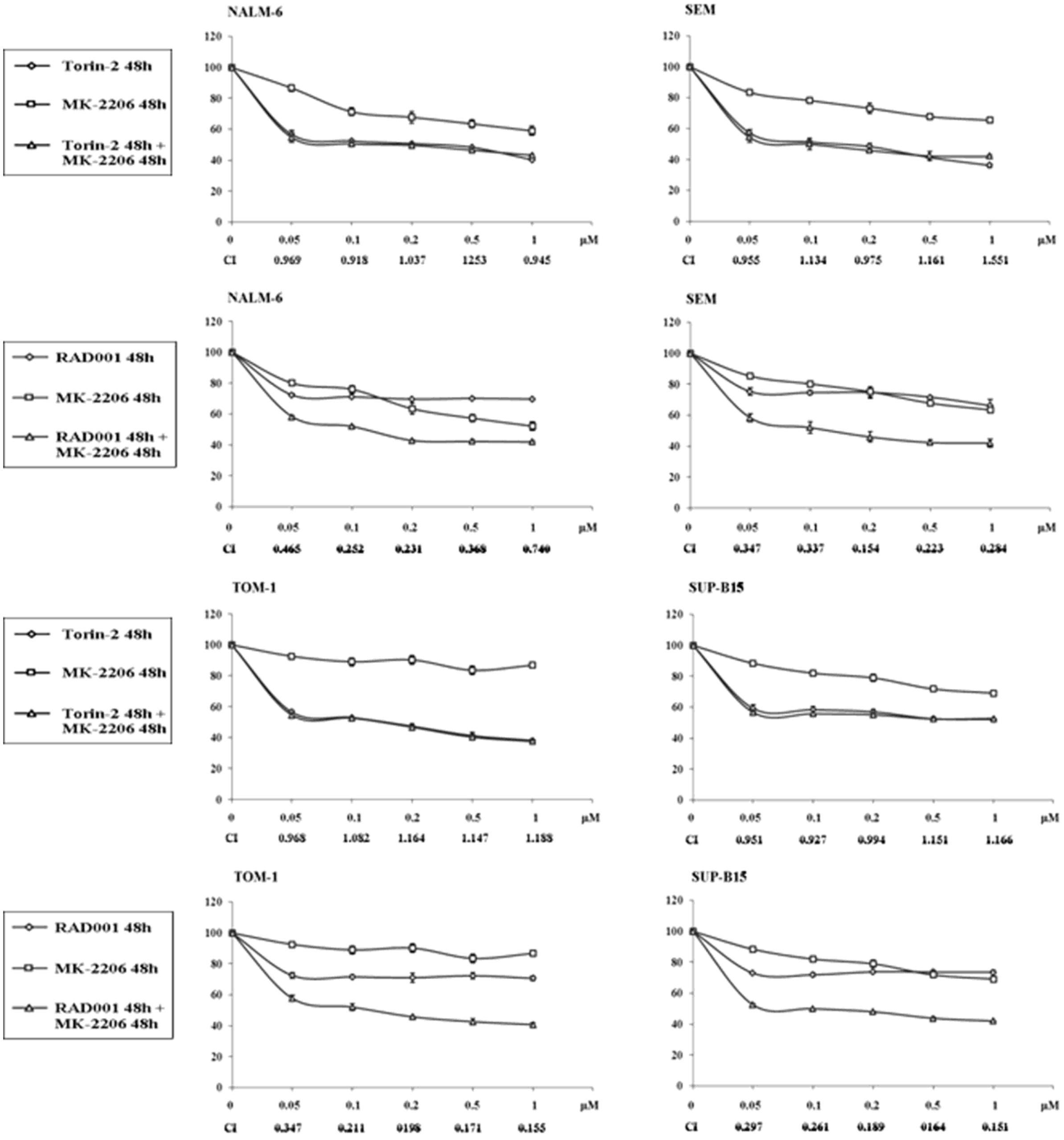

Supplementary Figure 1: NALM-6, SEM, TOM-1 and SUP-B15 cell lines were treated with Torin-2 or RAD001, either alone or in combination with MK-2206 for 48h. Results are the mean of three different experiments ± SD. Combination index (CI) value for each data point was calculated with the appropriate software for dose effect analysis (Calcusyn).
